# Remarks on a Paper Entitled "Medical Topography of New Orleans, &c." in the Edinburgh Journal

**Published:** 1816-08

**Authors:** 

**Affiliations:** one of the Editors


### *Remarks on a Paper entitled "Medical Topography of New Orleans, &c. in the Edinburgh Journal.*

By Mr. Johnson, one of the Editors.

THE Edinburgh Journal for April 1816, opens with a long, and by no means uninteresting communication, on the Medical Topography of New Orleans, with an account of the diseases that affected our fleet and army on the late expedition against that city; by an anonymous author. The stream of good sense, reading, and observation, however, is, like the Mississipi which he describes, rendered too often *turbid;* and that by pedantry and affectation.

The substance of the medical topography conveys only this intelligence, that the Mississipi winds through a level, low, marshy plain, from which abundance of paludal effluvia issues in the warm season particularly, giving rise in conjunction with aerial vicissitudes, in winter to dysentery, and in summer to marsh fevers of a very bad description.

A communication from Mr. Boyd, in the Medico-Chirurgical Journal for February last, shewed that the plan of treating dysentery by *scruple doses of calomel,* had been successfully pursued by him; and he there acknowledges that he first adopted the practice from perusing my work on Tropical Climates; *vide page* 117. The writer in the Edinburgh Journal, accuses me of promulgating this practice as a novelty, when it was long known to my brethren in the navy who had practiced in tropical climates."

Now, I defy him, or any other man, to shew a single written or printed document, in proof of this assertion; for previously to the publication of my work, I searched the whole of the Naval Medical Journals at the Transport Office, without meeting with a hint of the kind, but those which I have stated in my work; and these were contemporary with my own practice in India.

I am not anxious, however, about any merit in first promulgating the utility of calomel in *large doses,* for the cure of dysentery; but I wish to establish the fact by as many authorities as possible. I shall, therefore, bring forward my accuser himself. After some preliminary steps, he says, "I immediately commenced the use of calomel, and *pushed on boldly to salivation,* (a sentence, by the bye, copied literally from my work) from the belief which seems to be well founded, of an occult connection betwixt dysentery and a morbid condition of the liver." Again, "one scruple night and morning was the most usual prescription." "I have given a scruple night and morning so often, that I have long ceased to be at all anxious about hypercatharsis. It certainly seldom, in any case, increases the tormina or tenesmus; but generally lessens both very materially."—In respect to the combination of opium with calomel in dysentery, this well-informed writer coincides with what I have urged in my work; and states, that—"Candour obliges him to say that he has used it largely, and never noticed any of the unfavourable effects urged against it." 137.

Having shewn, I hope on practical grounds, the utility of mercury as a Sialagogue, *after* copious depletion in fevers of the East, the writer of the article in question, makes the following sarcastic allusion to my plan. "An attempt has lately been made to *clap up a match* [a very elegant expression for so classical a writer] betwixt the depletory and mercurial methods, and to call in the aid of both in the same case. The most respectable, if not the original proposer of this *incongruous* union, is Mr. Johnson, in his valuable work before referred to. What table of affinities suggested this coalition, it would be in vain to conjecture." 155.

This very shrewd Gentleman will probably be a little surprised, when I make *himself* furnish this same table of affinities that clearly indicates the ridiculed coalition. At page 137, he makes the following assertion:—"Of those who have lived for any length of time within the Tropics, it will be found that *four-fifths* have one viscus or other *in the abdomen*, more or less altered by morbid action."—Now, that the liver and spleen are the organs principally affected, needs not be insisted on; and if so, when fever attacks such persons, is not the influence of mercury clearly indicated, after the vascular system is unloaded by venæsection, and the gastric system cleared by purgatives, as recommended from extensive observation in my work? Where, now, is this incongruous union?—"Calomel," says he, "that great specific in obstructions of the liver, given in large doses, combined with opium, to cause it to be retained in the system, corrects the condition of the liver, prompts healthy secretion, *and resolves pyrexia.*" 143. With inexplicable inconsistency, this gentleman, a few pages further on, says, that "it is a matter of extreme uncertainty, whether mercurials can find their way into the system, *until the paroxysm of fever is dissolved.* Its action, even were it absorbed, would be rather hurtful." 156.—Thus the same medicine which, in one breath, "resolves pyrexia," in another, "is rather hurtful, or is incapable of entering the system during pyrexia!

But I have a more serious charge against the writer in the Edinburgh Journal. His frequent allusions to my work, prove him acquainted with its contents; yet, after accusing me of laying claim to novelty in the practice of dysentery which was not my own, he inserts a long passage at page 142, which is almost literally copied from my work, and for which he claims *originality!* Speaking of the influence which the liver exerts on other organs, he observes—"Its secretions influence the state of the stomach, and are influenced in their turn by the passions of the mind; and many facts would lead us to believe, that there is a *hitherto undescribed* sympathy betwixt *this* organ and the *brain.*" This gentleman must have read several pages in my work, expressly delineating this "*hitherto undescribed* sympathy," which he modestly places to his own credit.

I can assure the gentleman in question, that my observations on his paper are not influenced either by his praises or censures on my work; but from a sincere wish that truth may prevail, and that practical facts may not be sacrificed at the altar of ridicule or prejudice. The gentleman never practised in the eastern world, where hepatic affections are so conspicuous in all febrile states of the system, and consequently, where mercury is so necessary after proper depletion. He ought not, therefore, to have treated the observations of those who have had experience there, with such levity. In most other respects, I perfectly coincide with his ideas expressed in this paper; and am convinced, that with more experience and maturity of judgment, he *will yet* evince himself a m[illegible]n of very considerable *talent;* of which title he will probably be more proud than he is now of *learning.*

P. S. The author of the above-mentioned paper, quotes the authority of Mr. Boyd, Surgeon of the Hospital Ship at New Orleans, and justly characterizes him as "a gentleman of uncommon ability and experience in every department of the Profession." The same Mr. Boyd, in the February number of this Journal, speaking of West India fever, remarks, "although mercury is objected to by many practitioners of acknowledged abilities and experience, I have generally found, *after premising the necessary evacuations,* that mercury had the happiest effects, when judiciously administered, namely, in re-establishing all the healthy secretions and excretions."—*Med. Chir. Journal,* vol. i. p. 113.

Here, then, is this "*incongruous union*" maintained by a man whom he eulogises. Mr. Boyd first tried calomel in scruple doses in dysentery, at Mahon, from my recommendation of it; he afterwards introduced it at New Orleans, among the Naval Surgeons there; and the writer of the paper seeing the plan practised by Mr. Boyd, very coolly accuses Mr. Johnson of claiming the novelty of "a practice long known to his brethren in the Navy."

This gentleman ranges himself among the anti-contagionists, or anti-bulam party. It is curious to see in the same Journal two gentlemen from the same spot (Gibraltar) maintaining different sides of the question! Dr. Pym, in his work on the Bulam Fever, points out "the endemic of Batavia" in my work, as affording a specimen of the Bulam, and suggests its introduction into Java from Siam. But finding afterwards, that it rather *inconvenienced* his favourite opinion, he gravely sets about proving (in the February number of this Journal) that the Batavian Endemic was not at all the Bulam, which he before admitted it to be! After such specimens of *party,* it is needless to expect unbiassed discussions on litigated points, either of doctrine or practice!

